# Metagenomic and functional analyses of the consequences of reduction of bacterial diversity on soil functions and bioremediation in diesel-contaminated microcosms

**DOI:** 10.1038/srep23012

**Published:** 2016-03-14

**Authors:** Jaejoon Jung, Laurent Philippot, Woojun Park

**Affiliations:** 1 Laboratory of Molecular Environmental Microbiology, Department of Environmental Science and Ecological Engineering, Korea University, Seoul, 02841, Republic of Korea; 2 INRA Dijon, UMR 1347 Agroecologie, Dijon, France

## Abstract

The relationship between microbial biodiversity and soil function is an important issue in ecology, yet most studies have been performed in pristine ecosystems. Here, we assess the role of microbial diversity in ecological function and remediation strategies in diesel-contaminated soils. Soil microbial diversity was manipulated using a removal by dilution approach and microbial functions were determined using both metagenomic analyses and enzymatic assays. A shift from *Proteobacteria*- to *Actinobacteria*-dominant communities was observed when species diversity was reduced. Metagenomic analysis showed that a large proportion of functional gene categories were significantly altered by the reduction in biodiversity. The abundance of genes related to the nitrogen cycle was significantly reduced in the low-diversity community, impairing denitrification. In contrast, the efficiency of diesel biodegradation was increased in the low-diversity community and was further enhanced by addition of red clay as a stimulating agent. Our results suggest that the relationship between microbial diversity and ecological function involves trade-offs among ecological processes, and should not be generalized as a positive, neutral, or negative relationship.

The relationship between biodiversity and ecosystem function has long been of interest in ecology^1^. To address this fundamental question, many studies have been performed in natural environments using biodiversity gradients^2^ or experimental manipulation of the ecosystem^3^. A positive relationship between plant biodiversity and plant productivity was reported for experimentally manipulated grasslands^4^. Microcosm studies using assemblages of different genotypes or species of bacteria also revealed a positive relationship between diversity and productivity or bacterial respiration^5^,^6^. However, previous studies on the role of microbial biodiversity have focused on relatively few processes, such as denitrification, primary production, or organic carbon degradation^7^,^8^,^9^. Although these studies can provide valuable data on biodiversity and ecological function, limited analysis of other functions that might be supported by microbial biodiversity has been performed.

Anthropogenic disturbances, such as contamination with heavy metals^10^ and hydrocarbons^11^, can affect microbial community structure and biodiversity. In general, hydrocarbon contamination has been shown to reduce microbial biodiversity^12^, as hydrocarbons are toxic to microbial cells and have adverse effects on the cell membrane^13^. However, hydrocarbons can be used as a carbon source by some species of microbes^14^, and their presence can therefore enrich certain microbial populations. Most studies conducted at hydrocarbon-contaminated sites have focused on bioremediation efficiency and shifts in microbial community structure, with little attention paid to the resulting changes in other ecosystem functions^15^. However, according to the “biological insurance” hypothesis, there is a greater probability that a species that can tolerate or degrade a pollutant would be present in a high-diversity community than in a low-diversity community. Girvan *et al.*^16^ showed that highly diverse soil microbial communities are more resistant to benzene perturbation than those with low diversity are. However, other studies have reported no connection between biodiversity and resistance or resilience to heat or copper perturbations^17^,^18^. This discrepancy among results regarding biodiversity and community stability can perhaps be explained by the different functions investigated.

Here, we assess the roles played by microbial diversity in soil function in natural and diesel-contaminated soils. We manipulated microbial diversity using a removal by dilution approach and performed metagenomic analysis to investigate the genetic basis of a variety of processes; additionally, we performed several enzyme assays. To assess the impact of loss of biodiversity on bioremediation, we spiked red clay and processed red clay with diesel, as it is a biostimulant^19^. Processed red clay is produced from red clay using a patented method (Patent# 0886082 in the Republic of Korea); it promotes diesel biodegradation and alters microbial community structure.

## Results and Discussion

### Bacterial abundance after dilution and soil recolonization

**Figure 1:**
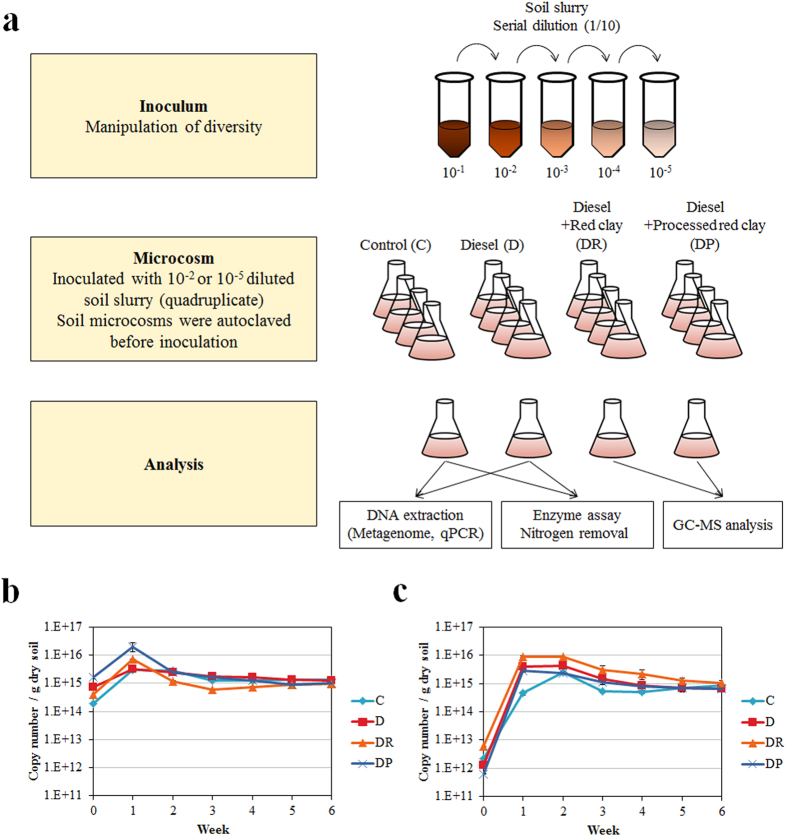
Schematic description of experimental design and quantitation of microbial communities in microcosms based on 16S rRNA gene copy number. (**a**) Soil was serially diluted and inoculated to sterilized soil slurry microcosms subjected to metagenomic analysis and experiments. (**b**) Copy number of the 16S rRNA gene in the 10^−2^-inoculated samples and (**c**) 10^−5^-inoculated samples. C, control; D, diesel-spiked soil; DR, diesel-spiked soil and red clay; DP, diesel-spiked soil and processed red clay.

Bacterial communities were quantified using the 16S rRNA gene copy number as a molecular marker (Fig. 1). At week 0, the 16S rRNA gene copy number was 1.88 ± 0.14 × 10^14^ to 1.64 ± 0.17 × 10^15^/g dry soil and 5.99 ± 0.03 × 10^12^ to 6.30 ± 0.26 × 10^12^/g dry soil for the microcosms inoculated with the 10^−2^ and 10^−5^ dilutions, respectively. The difference between the 16S rRNA gene copy numbers of the 10^−2^- and 10^−5^-inoculated samples was approximately 10^3^, consistent with the difference in dilution factors. Following a transient increase at week 1, the 16S rRNA gene copy number in 10^−2^-inoculated samples reached approximately 1.0 × 10^15^ after 6 weeks; no significant differences were observed between treatments. It should be noted that diesel-spiked samples showed a higher 16S rRNA gene copy number than controls during incubation (Fig. 1). The increase in community size observed in diesel-spiked soil is consistent with the results of a previous study^19^; the diesel supplied an alternative carbon source to soil organic matter. After 6 weeks, the 10^−2^- and 10^−5^-inoculated samples showed identical bacterial abundance. Different dilutions of microbial samples have previously been shown to have similar capacity to colonize sterile soil^20^.

### Microbial community structure differs at different levels of diversity

**Figure 2:**
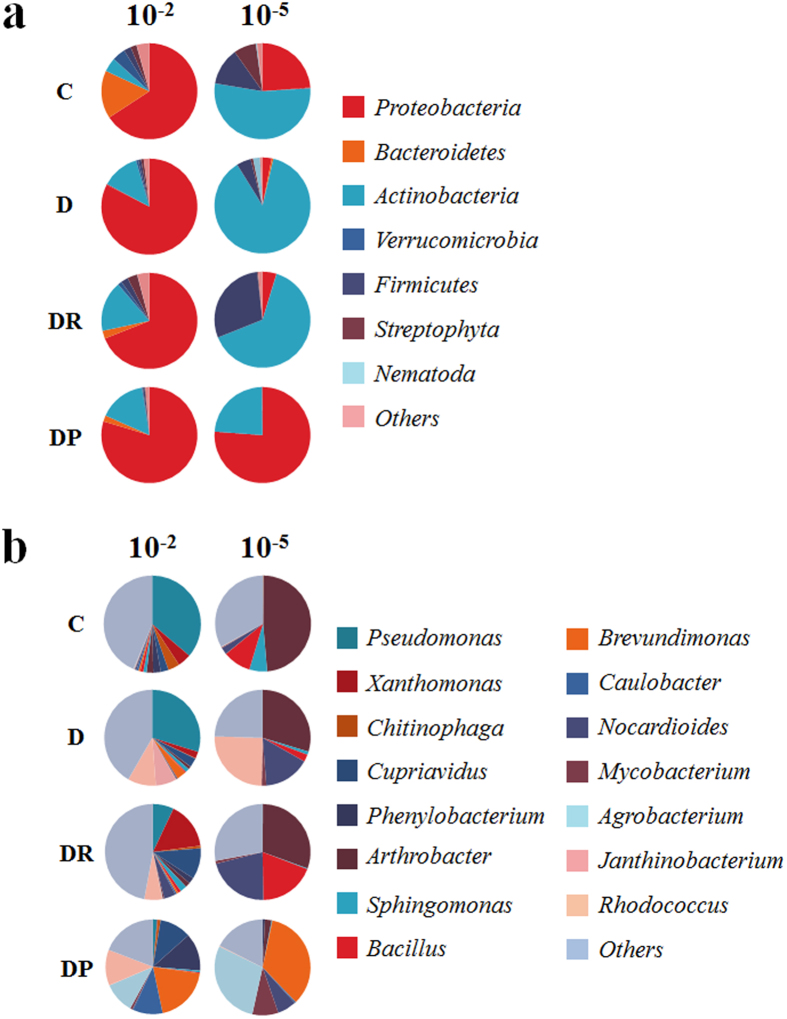
Analysis of microbial communities using rRNA gene sequences and MG-RAST. Taxa with abundance <5% are presented as “Others.” (**a**) Phyla (**b**) Genera C, control; D, diesel-spiked soil; DR, diesel-spiked soil and red clay; DP, diesel-spiked soil and processed red clay.

The alpha diversity calculated using MG-RAST showed that, in the control treatment, the 10^−2^-inoculated samples contained a higher number of OTUs (268 OTUs) than the10^−5^-inoculated samples (99 OTUs) (Supplementary Fig. S1). The presence of diesel in the soil further decreased the number of OTUs to 162 and 51 in the 10^−2^- and 10^−5^-inoculated microcosms, respectively. The reduction in diversity produced by hydrocarbon contamination is consistent with the results of previous studies^12^. This could be attributed to the toxicity of diesel, as shown in studies based on single strains and communities of bacteria^11^,^21^. The soil sample used in this study was the identical sample described in our previous study^19^. According to the previous community analysis of soil without any treatment or incubation, *Proteobacteria* is the predominant phylum followed by *Acidobacteria* and *Actinobacteria* and their relative abundances were 50%, 13%, and 10%, respectively. Predominant families were *Rhodospirillaceae* (9%), *Comamonadaceae* (7%), and *Bradyrhizobiaceae* (7%). Serial dilution approach would have removed minor taxa comprising less than 1% relative abundance. Taxonomic analysis of the bacterial community using 16S rRNA gene sequences from the metagenomic dataset showed that *Proteobacteria* was the most abundant phylum in all 10^−2^-inoculated microcosms, whereas all 10^−5^-inoculated microcosms, except the DP5 treatment, were dominated by *Actinobacteria* (Fig. 2a). In contrast, Tardy *et al.*^22^, who also used a removal by dilution approach to manipulate bacterial diversity, found that the abundance of *Proteobacteria* increased with increasing dilutions. This discrepancy can be explained by differences in soil type, since the abiotic properties of the soil will influence the establishment and survival of inoculated strains, known as the “habitat filtering” effect. The soil type will also determine the soil microbial seed bank used for inoculation.

In addition to its effect on the alpha diversity, diesel also appeared to affect the dominance of certain phyla, with *Proteobacteria* and *Actinobacteria* being more abundant in diesel-spiked microcosms. These taxa are commonly found in both pristine^23^ and diesel-contaminated soil^12^, and a large proportion are potentially able to degrade alkanes, a major component of diesel^24^. Given their prevalence and alkane-degrading capacity, the reduction in microbial diversity caused by the addition of diesel, which is toxic to many microbes, might have provided the preferred conditions for proliferation of *Proteobacteria* and *Actinobacteria*^12^. Thus, the relative abundance of *Proteobacteria* and *Actinobacteria* increased from 70.5% and 77.3% in C2 and C5 to 95.2% and 90.6% in D2 and D5, respectively. *Actinobacteria* was the second-most abundant phylum in D2, DR2, and DP2, whereas *Bacteroidetes* was the second-most abundant group in C2. A Shift from *Proteobacteria*- to *Actinobacteria*-dominant community of 10^−2^- and 10^−5^-inoculated sample, respectively, was mainly due to increase in abundance of genus *Arthrobacter*, *Rhodococcus*, and *Nocardioides* which were known as hydrocarbon degraders^25^,^26^,^27^. More specifically, *Pseudomonas* and *Arthrobacter* were the predominant genera in C2 and C5, respectively (Fig. 2b). *Arthrobacter*, *Rhodococcus*, and *Nocardioides* were identified in D5. When diesel was present in red clay or processed red clay, the relative abundances of *Xanthomonas* (DR2), *Cupriavidus* (DR2 and DP2), *Phenylobacterium* (DP2), *Brevundimonas* (DP2), and *Caulobacter* (DP5) were higher than in C2. We observed increases in the relative abundance of *Brevundimonas* and *Agrobacterium* in DP2 and DP5. Processed red clay promoted the abundance of these two genera of *Alphaproteobacteria*, resulting in *Proteobacteria*-dominant communities in DP2 and DP5, regardless of the dilution factor of the initial inoculum. Community structure has often been reported to shift following hydrocarbon contamination^12^,^19^,^28^. The change in community profile has usually been explained by differing environmental conditions. For example, phylogenetic analysis of the bacteria present in contaminated soils showed that the dominance of a bacterial group depended on temperature and soil organic matter content^15^,^28^. However, our data demonstrate that biodiversity can also be a determining factor in shaping community structure when hydrocarbon contamination occurs.

### Changes in community diversity and structure result in differences in functional gene abundance

The results of taxonomic binning of functional genes were consistent with the results of our community analysis based on rRNA sequences. For example, a greater proportion of *Actinobacteria*-affiliated genes were observed in the 10^−5^-inoculated samples (Supplementary Fig. S2). PCA based on phylum-level community structure (Supplementary Fig. S3A) and functional annotation of metagenomic data (KO level 3) (Supplementary Fig. S3B) showed that structurally similar communities are also functionally similar; suggesting that changes in community membership resulting from diversity manipulation also brought functional changes. The abundance of various functional genes, as determined by metagenomic sequence analysis, was compared using STAMP. Of the 140 KO categories analyzed, 51, 74, 46, and 43 differed significantly for the C, D, DR, and DP treatments, respectively, between 10^−2^- and 10^−5^-inoculated microcosms (Welch’s two-sided t-test, *p* <0.05). This linkage between function and community structure has previously been described in soil ecosystems. For example, catabolic capability was highly associated with copiotrophic and oligotrophic taxa, which dominate areas of high and low nitrogen fertilization, respectively^29^. We observed that some functions that are performed by all bacteria, such as amino acid metabolism and nucleotide metabolism, did not differ between samples. However, specific functions that are only performed by certain groups of bacteria, such as hydrocarbon degradation and tetracycline biosynthesis, were significantly affected by changes in community diversity and composition. Susceptibility of some functions to loss of diversity was previously reported from heavy metal-contaminated soil^30^; the susceptibility was attributed to the fact that these functions are performed by a narrow group of specialized species. According to the orthologue richness reduction model, loss of species richness must be <10% to avoid significant loss of ecosystem services^28^. Given the number of OTUs identified in the samples, 10^−5^-inoculated samples might have lost a subset of species that perform a specific ecological function. The different clustering of 10^−5^-inoculated samples on the PCA plot suggested that the functions that remained would differ by treatment, for example between the two types of red clay contaminated with diesel.

### Effect of microbial community manipulation on alkane degradation

**Figure 3:**
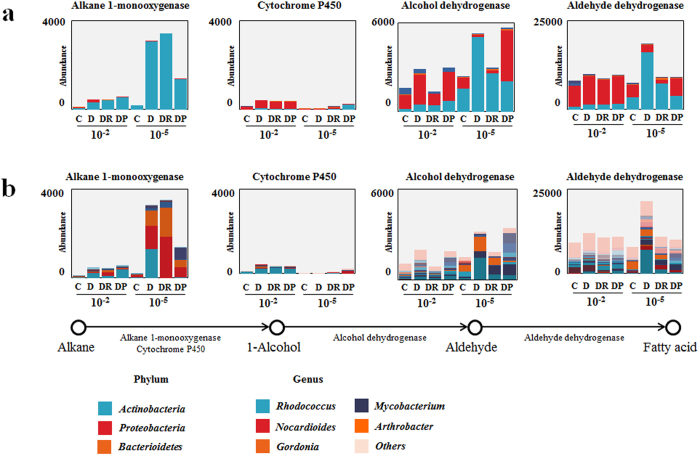
Genes related to alkane oxidation in microcosms of differing biodiversity. Gene abundance and taxonomic affiliation at the phylum level were determined using the KEGG Orthology and M5NR databases, respectively, from MG-RAST. Taxa with abundance <5% are presented as “Others.” C, control; D, diesel-spiked soil; DR, diesel-spiked soil and red clay; DP, diesel-spiked soil and processed red clay. (**a**) phylum (**b**) genus.

**Figure 4:**
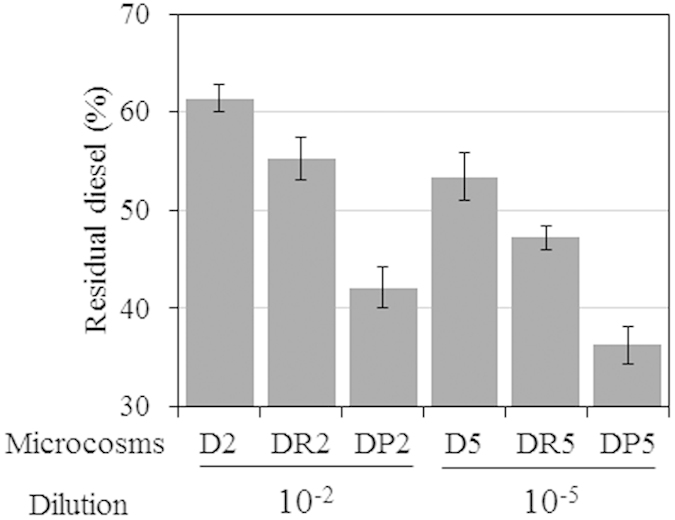
Diesel biodegradation in microcosms after 6 weeks. Residual percentages were calculated based on the major diesel components (alkane chain length C9–C20). D, diesel-spiked soil; DR, diesel-spiked soil and red clay; DP, diesel-spiked soil and processed red clay.

To assess the effect of diversity loss on the potential of the communities present in the different soils to degrade diesel, genes related to alkane oxidation, such as those encoding alkane monooxygenase, cytochrome P450, alcohol dehydrogenase, and aldehyde dehydrogenase, were analyzed (Fig. 3). Our results showed that the reduction in microbial community diversity did not always lead to a decrease in functional gene abundance. Thus, the abundance of the alkane monooxygenase gene was 2.1–13.1-fold higher in low-diversity communities. Previous studies showed that *alkB* abundance generally increases in alkane-contaminated soils^31^,^32^,^33^; however, the appearance of various primary alkane-degrading species has not been discussed in terms of the reduction of diversity. The abundance of cytochrome P450 genes did not differ significantly between treatments, except for D2 and D5. Alkane monooxygenase and cytochrome P450 were associated mainly with *Actinobacteria*. At the genus level, alkane 1-monooxygenase was taxonomically affiliated with *Rhodococcus*, *Nocardioides*, and *Gordonia.* Investigation of the taxonomic affiliations of the alcohol and aldehyde dehydrogenases revealed that *Proteobacteria* and *Actinobacteria* predominated in the 10^−2^- and 10^−5^-inoculated microcosms, respectively. However, no significant changes in the abundances of alcohol dehydrogenase or aldehyde dehydrogenase genes were observed. Alcohol dehydrogenase and aldehyde dehydrogenase are expected to be also present in bacteria other than *Proteobacteria* or *Actinobacteria* because these enzymes degrade various forms of alcohols and aldehydes, respectively. The major component of the diesel used in this study was alkanes, with chain lengths of C9–C20; GC-MS analysis showed that levels of aromatic hydrocarbons and alkanes with longer chains (C21–C24) were very low. Therefore, the efficiency of diesel biodegradation was estimated based on the concentration of alkanes (C9–C20). After 6 weeks, the concentration of diesel was determined. The presence of red clay or processed red clay enhanced biodegradation of alkanes, as described previously^19^ (Fig. 4). Alkane biodegradation was more efficient in low-diversity microbial communities (10^−5^-inoculated samples) than in 10^−2^-inoculated microcosms in the same treatment group. This result is consistent with the results of the metagenomic analysis, which showed that alkane monoxygenase and P450 genes were more abundant in low-diversity communities. Alkanes can be degraded by diverse types of bacteria, such as species of *Pseudomonas*, *Rhodococcus*, and *Arthrobacter*^26^,^28^,^30^. *Pseudomonas* and *Arthrobacter* were dominant in the 10^−2^- and 10^−5^-inoculated samples, respectively. However, it was previously reported that the percentage of *alkB*- or cytochrome P450-containing genomes was higher for *Actinobacteria* (30.7% and 6.1%, respectively) than for *Proteobacteria* (13.3% and 3.6%, respectively)^11^. Therefore, it can be hypothesized that the higher efficiency of alkane biodegradation observed in low-diversity communities is due to the higher competitiveness of *Actinobacteria* in the less-diverse microcosms, resulting in a higher proportion of strains carrying *alkB*. In contrast, the DP treatment, which exhibited the highest level of diesel biodegradation, was dominated by *Proteobacteria* and most often showed lower abundance of the genes involved in diesel biodegradation. This suggests that the enhancement of diesel biodegradation resulted not only from alteration of community structure but other effects as well, for example, promotion of bacterial growth^19^ and upregulation of expression of genes related to alkane degradation and oxidative stress defense, as shown in our previous research^34^. It should be noted that the processed red clay used in this study did not contain organic nutrients, or nitrogen or phosphate fertilizers, and thus differed from the typical nutrient-rich soil used in traditional biostimulation strategies^35^. In fact, the DP5 sample, which contained processed red clay, showed differences in community structure and contained a key alkane degrader not present in other 10^−5^-inoculated samples. We recently reported that addition of processed red clay affects the expression of genes related to alkane metabolism, oxidative stress defense, and membrane fatty acid composition^35^, as well as community structure^19^. However, the detailed effects of processed red clay are only beginning to be understood.

Low-diversity communities have been reported to show a higher efficiency of alkane biodegradation^36^. In their study, gentamicin and vancomycin, which target gram-negative and -positive bacteria, respectively, were used to reduce microbial diversity^36^. Biodegradation was most effective in low-diversity communities and communities with low 16S rRNA gene copy numbers. Collectively, these results suggest that certain ecological functions, such as alkane biodegradation, do not occur optimally in more diverse microbial communities and that shifts in the abundance of taxa can produce conditions that are optimal for alternative ecological functions.

### Effect of microbial community manipulation on biogeochemical cycling

**Figure 5:**
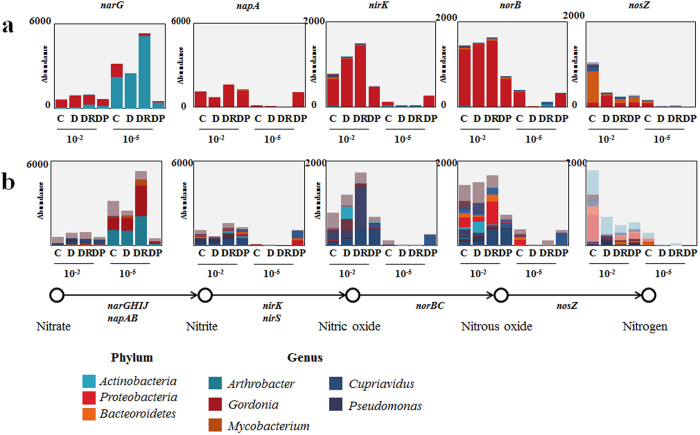
Abundance of genes related to denitrification in microcosms of differing biodiversity and microbial community structure. Taxa with abundance <5% are presented as “Others.” (**a**) phylum (**b**) genus C, control; D, diesel-spiked soil; DR, diesel-spiked soil and red clay; DP, diesel-spiked soil and processed red clay.

**Figure 6:**
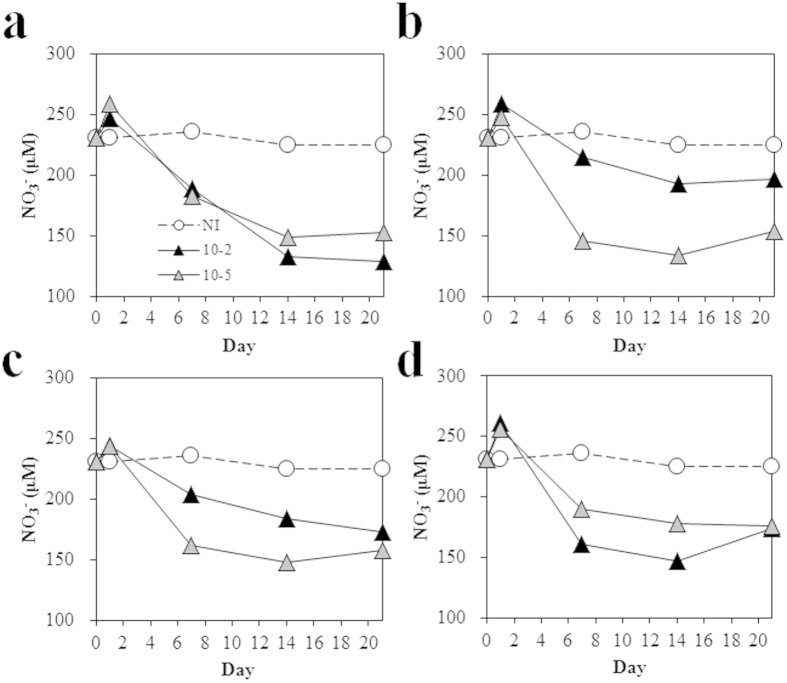
Nitrate reduction by microbial communities in microcosms of differing biodiversity and varying structure. (**a**) C2 and C5 (**b**) D2 and D5 (**c**) DR2 and DR5 (**d**) DP2 and DP5. NI indicates no inoculation (sterile soil) to show that reduction of nitrate was performed by biological processes.

We first focused on the nitrogen cycle because nitrogen is a key nutrient that may be vulnerable to environmental perturbations, according to previous studies performed in polluted areas or experimentally manipulated conditions^11^,^37^. We assessed how N-cycling was affected by diversity loss by analyzing the relative abundances of N cycle-related genes from our metagenomic data. We found lower abundances of the *napA*, *nirK*, *norB*, and *nosZ* denitrification genes in the 10^−5^-inoculated samples than in the 10^−2^-inoculated samples. In contrast, the abundance of *narG*, which encodes the nitrate reductase that catalyzes the first step of the denitrification and DNRA (dissimilatory nitrate reduction to ammonium) processes, was greater in the 10^−5^-inoculated samples than in the 10^−2^-inoculated samples, except in the case of DP5 (Fig. 5). Most of the *narG* genes identified were found to belong to *Actinobacteria*, which supports the results of our taxonomic analyses of the bacterial community and suggests that nitrate-reducing *Actinobacteria* were selected during the soil recolonization process. To assess how these changes affected N cycling, the reduction of NO_3_^−^ to NO_2_^−^ was monitored. The concentration of nitrate decreased over time in all microcosms and D5 and DR5 showed a greater extent of nitrate reduction than D2 and DR2 (Fig. 6). Here, the experimental data only partially correspond with our metagenomic analysis of N cycle-related genes. This could be due to the fact that nitrate concentration is not only dependent on nitrate reduction by denitrification or DNRA but also on nitrate assimilation. We also investigated the effects of diversity loss on carbon, sulfur, and phosphate cycling by monitoring pectin utilization, along with β-galactosidase, arylsulfatase, and phosphatase activities. We identified differences in pectin utilization and in β-galactosidase and arylsulfatase activities among the various microbial communities; however, we could not identify any patterns among samples. Arylsulfatase activity did not differ significantly among samples (Supplementary Fig. S4). The abundance of genes encoding pectinesterase, β-galactosidase, arylsulfatase, and phosphatase was not correlated with the activity data, probably due to non-specific enzymatic reactions and the heteropolymeric characteristics of pectin. Although an attempt has been made to use soil enzyme activity as an indicator of hydrocarbon bioremediation^38^, contradictory results were obtained^39^. The absence of a general pattern in soil enzyme activity with hydrocarbon contamination and differences in diversity could be due to physiological complexity at the community level^40^. However, our metagenomic data and enzyme activity analyses suggested that community membership, as determined by community diversity, affected the nutrient cycle in a contaminated soil environment.

## Conclusion

Collectively, our results highlight that both community diversity and membership are key drivers of soil function. Thus, we showed that diesel biodegradation was enhanced in less-diverse soil microcosms due to the greater abundance of bacterial groups possessing genes involved in diesel biodegradation. However, loss of biodiversity resulted in decreased abundance of genes involved in reduction of nitrite, nitric oxide, and nitrous oxide. Genetic and experimental evidence clearly showed that biodiversity affected alkane biodegradation, denitrification, and nutrient cycling-related soil enzyme activity, in varying ways. Microbial taxa present at low abundance under natural conditions might gain a competitive advantage and thrive when initial biodiversity is impaired. Performance of specialized functions associated with a specific taxon results in a shift in the corresponding ecological function. Changes in community structure, diversity, and particular functions often occur in soils contaminated with other pollutants, such as heavy metals, and it would be revealing to implement the analyses employed in this study in various polluted environments^41^,^42^,^43^. Many previous studies drew hasty conclusions about the relationship between ecological function and biodiversity, based on alteration of one or two functions, while neglecting to examine others. The combination of metagenomic and enzymatic analyses performed in this study suggests that some functions might be strengthened while others were lost during shifts in the microbial community, and that such trade-offs between ecological functions were brought about by loss of diversity. This conclusion may also be relevant to the study of bioremediation, which often pursues a reduction in the total quantity of pollutants as the primary or only goal; due consideration should be given to ecological function during the bioremediation process. Overall, our results indicate that community membership should be taken into account when assessing the relationship between biodiversity and ecosystem function, highlighting process-specific responses to loss of biodiversity.

## Methods

### Manipulation of diversity in a diesel-contaminated microcosm supplemented with red clay

The soil used in this study was the same as that described previously^19^. Briefly, soil samples were collected from Bucheon (Kyonggi province, Republic of Korea; N 37°30′26.44, E 126°48′39.01). The soil had a sandy loam texture and 30% water content. Soil pH was 9.44 ± 0.22 and contained 13,846 ± 435 mg/kg of total organic carbon. Soils were sieved to achieve a particle diameter of <2 mm. The soil slurry microcosm was generated in a 100-mL flask containing 10 g of soil and 50 mL of distilled water (DW), which was autoclaved (Fig. 1a). The soil in the microcosms was the source of carbon and other nutrients. Microcosm experiments were performed in quadruplicate. After constructing the microcosms, we extracted DNA from the soil slurry using a NucleoSpin Soil kit (Macherey-Nagel, Germany), according to the manufacturer’s instructions. Soil slurries from the microcosms were centrifuged to obtain soil particles and microbial cells. Supernatants were discarded and pellets containing soil and cells were subjected to DNA extraction. The 16S rRNA gene was amplified using 27F (5′-AGAGTTTGATCMTGGCTCAG-3′) and 1492R (5′-CGGTTACCTTGTTACGACTT-3′) primers. PCR was performed at 95 °C for 90 s, followed by 35 cycles at 95 °C for 24 s, at 56 °C for 24 s, at 56 °C for 24 s, and a final extension step at 72 °C for 5 min using a Mastercycler thermal cycler (Eppendorf, Hauppauge, NY, USA). Additionally, we assessed β-galactosidase activity in autoclaved soil samples. All soil samples were PCR-negative and lacked β-galactosidase activity, indicating that microbial activity had been abolished. Microcosms lacking red clay were spiked with 0.1% (v/v) diesel, while those with red clay were spiked with 1% (v/v) diesel. Microcosms containing processed red clay were spiked with 0.1% (v/v) diesel. All red clay was autoclaved before use. These two types of red clay were previously shown to enhance diesel bioremediation^19^. To manipulate biodiversity, we employed a dilution strategy that has been described previously^3^. Experimental procedures are depicted in Fig. 1a. Intact soil (1 g) was mixed with 10 mL of sterile DW by vortexing for 10 min. Soil suspensions were serially diluted ten-fold. We used 1 mL of the 10^−2^ and 10^−5^ diluted suspensions to inoculate the autoclaved microcosms and examined four different treatments: no treatment (control); 0.1% (v/v) diesel, 1% (v/v) diesel and red clay; and 0.1% (v/v) diesel and processed red clay. We designated the control, diesel, diesel and red clay, and diesel and processed red clay groups as C, D, DR, and DP, respectively; we added a ‘2’ or ‘5’ to each group designation to indicate the dilution factor used. Microcosms were incubated at 20 °C with shaking at 150 rpm; the incubation temperature we used was within the temperature range of the sampling site.

### Gas chromatographic analysis of diesel

The composition of the diesel used for spiking of soil samples was analyzed using a gas chromatography equipped with a mass spectrometer (GC-MS) (Agilent 6890N gas chromatograph and 5975 mass spectrometer). A HP-5MS column was used. Diesel dissolved in dichloromethane (2 μL) was injected into the GC. The injector and detector were maintained at 280 °C and 300 °C, respectively. Oven temperature was held at 45 °C for 2 min and increased to 310 °C at a rate of 10 °C/min with an additional hold for 25 min. For chemical identification of peaks, results were searched against the MS spectral library (NIST05 library) with a score cutoff of >80%. Quantification of residual diesel after incubation of microcosms were performed using a GC equipped with a flame ionization detector (FIC), as previously described^19^.

### Quantification of bacterial community abundance

After inoculation, we monitored changes in microbial community size by quantitative PCR (qPCR), by determining the 16S rRNA gene copy number. For this purpose, the primers 341F and 534R were used to amplify the 16S rRNA gene^44^. A standard curve for 16S rRNA gene copy number has previously been constructed by our lab^45^. DNA extraction and PCR were performed as described above. PCR was performed using a CFX-96 PCR machine (Bio-Rad).

### Metagenomic sequence analysis

For the metagenomic sequencing analysis, DNA was isolated from duplicate soil slurry samples after 6 weeks, as described above. Generation of a sequence library was conducted using the MiSeq System (Illumina Inc., San Diego, CA, USA) at Macrogen Inc. (Seoul, Republic of Korea) following the 2 × 100-bp paired-end protocol. Sequencing data was processed using Illumina Pipeline (CASAVA) version 1.8.2. Paired-end reads that contained >80% of bases with a base quality ≥Q16 were assembled. The results of sequencing are summarized in Supplementary Table S1. Because the depth of sequencing differed between samples, assembled sequences were subsampled (5,375,854 reads, the minimum number of reads) and uploaded to MG-RAST^46^ for further analysis. Metagenomic sequences were deposited in MG-RAST (http://metagenomics.anl.gov/) under deposition numbers 4569530.3 to 4569537.3. Statistical analysis of the metagenomic sequencing data was performed using STAMP^47^ and R. Community structure was analyzed based on ribosomal RNA sequences from the metagenomic data using the Best Hit Classification function and the M5RNA database available in MG-RAST. To identify the taxonomic affiliation of functional genes, selected genes were stored in Workbench and then subjected to Best Hit Classification using the M5NR database. The cutoff for metagenomic analysis was a maximum e-value of 1e-5, a minimum identity of 60%, and a minimum alignment length of 15 bp.

### Measurement of inorganic nitrogen compounds

Another set of microcosms were used to monitor concentrations of nitrates and nitrites. These microcosms contained autoclaved soil slurry and were inoculated from microcosms that had been incubating at 20 °C for 6 weeks. The caps of the bottles were tightly sealed and helium gas was used to replace the gas in the bottle headspace. The concentrations of nitrates and nitrites in the soil slurry were measured using an ion chromatograph equipped with an autosampler (Dionex AS40, USA). The mobile phase (DW) passed through the column at a flow rate of 1.0 mL/min. The sample injection volume was 5 mL. The suppressor was generated using 20 mM potassium hydroxide. A standard curve was established using nitrate and nitrite standard solutions (Kanto Chemical, Japan).

### Assays for determining various ecological functions

After 6 weeks, soil slurry microcosms were tested to identify differences in various ecological functions. To determine their ability to utilize pectin, soil slurry samples were transferred to sterile minimal salt basal media^48^ supplemented with pectin. Utilization of pectin was assessed through the determination of increased protein content after 1 week. We determined the activities of β-galactosidase, phosphatase, and arylsulfatase as described previously^49^,^50^,^51^.

## Additional Information

**How to cite this article**: Jung, J. *et al.* Metagenomic and functional analyses of the consequences of reduction of bacterial diversity on soil functions and bioremediation in diesel-contaminated microcosms. *Sci. Rep.* **6**, 23012; doi: 10.1038/srep23012 (2016).

## Supplementary Material

Supplementary Information

## References

[b1] Sutherland W. J. *et al.* Identification of 100 fundamental ecological questions. J Ecol 101, 58–67 (2013).

[b2] Balvanera P. *et al.* Quantifying the evidence for biodiversity effects on ecosystem functioning and services. Ecol Lett 9, 1146–1156 (2006). 16972878 10.1111/j.1461-0248.2006.00963.x

[b3] Philippot L. *et al.* Loss in microbial diversity affects nitrogen cycling in soil. ISME J 7, 1609–1619 (2013). 23466702 10.1038/ismej.2013.34 PMC3721106

[b4] Wu J. *et al.* Testing biodiversity-ecosystem functioning relationship in the world’s largest grassland: overview of the IMGRE project. Landscape Ecol 10.1007/s10980-015-0155-y (2015).

[b5] Hodgson D. J., Rainey P. B. & Buckling A. Mechanisms linking diversity, productivity and invasibility in experimental bacterial communities. Proc Biol Sci 269, 2277–2283 (2002). 12427320 10.1098/rspb.2002.2146 PMC1691149

[b6] Bell T., Newman J. A., Silverman B. W., Turner S. L. & Lilley A. K. The contribution of species richness and composition to bacterial services. Nature 436, 1157–1160 (2005). 16121181 10.1038/nature03891

[b7] Pedler B. E., Aluwihare L. I. & Azam F. Single bacterial strain capable of significant contribution to carbon cycling in the surface ocean. Proc Natl Acad Sci USA 111, 7202–7207 (2014). 24733921 10.1073/pnas.1401887111 PMC4034236

[b8] van der Heijden M. G. A. *et al.* Mycorrhizal fungal diversity determines plant biodiversity, ecosystem variability and productivity. Nature 396, 69–72 (1998).

[b9] Hou L. *et al.* Effects of sulfamethazine on denitrification and the associated N_2_O release in estuarine and coastal sediments. Environ Sci Technol 49, 326–333 (2015). 25525860 10.1021/es504433r

[b10] Pessoa-Filho M. *et al.* Microbiological functioning, diversity, and structure of bacterial communities in ultramafic soils from a tropical savanna. Antonie Van Leeuwenhoek 107, 935–949 (2015). 25616909 10.1007/s10482-015-0386-6

[b11] van Dorst J., Siciliano S. D., Winsley T., Snape I. & Ferrari B. C. Bacterial targets as potential indicators of diesel fuel toxicity in subantarctic soils. Appl Environ Microbiol 80, 4021–4033 (2014). 24771028 10.1128/AEM.03939-13 PMC4054227

[b12] Sutton N. B. *et al.* Impact of long-term diesel contamination on soil microbial community structure. Appl Environ Microbiol 79, 619–630 (2013). 23144139 10.1128/AEM.02747-12 PMC3553749

[b13] Sikkema J., de Bont J. A. & Poolman B. Mechanisms of membrane toxicity of hydrocarbons. Microbiol Rev 59, 201–222 (1995). 7603409 10.1128/mr.59.2.201-222.1995 PMC239360

[b14] Atlas R. M. Microbial degradation of petroleum hydrocarbons: an environmental perspective. Microbiol Rev 45, 180–209 (1981). 7012571 10.1128/mr.45.1.180-209.1981 PMC281502

[b15] Bell T. H. *et al.* Predictable bacterial composition and hydrocarbon degradation in Arctic soils following diesel and nutrient disturbance. ISME J 7, 1200–1210 (2013a). 23389106 10.1038/ismej.2013.1 PMC3660676

[b16] Girvan M. S., Campbell C. D., Killham K., Prosser J. I. & Glover L. A. Bacterial diversity promotes community stability and functional resilience after perturbation. Environ Microbiol 7, 301–313 (2005). 15683391 10.1111/j.1462-2920.2005.00695.x

[b17] Griffiths B. S. *et al.* An examination of the biodiversity–ecosystem function relationship in arable soil microbial communities. Soil Biol Biochem 33, 1713–1722 (2001).

[b18] Wertz S. *et al.* Decline of soil microbial diversity does not influence the resistance and resilience of key soil microbial functional groups following a model disturbance. Environ Microbiol 9, 2211–2219 (2007). 17686019 10.1111/j.1462-2920.2007.01335.x

[b19] Jung J., Choi S., Hong H., Sung J. S. & Park W. Effect of red clay on diesel bioremediation and soil bacterial community. Microb Ecol 68, 314–323 (2014). 24743885 10.1007/s00248-014-0420-7

[b20] van Elsas J. D. *et al.* Microbial diversity determines the invasion of soil by a bacterial pathogen. Proc Natl Acad Sci USA 109, 1159–1164 (2012). 22232669 10.1073/pnas.1109326109 PMC3268289

[b21] Kang Y. S. & Park W. Protection against diesel oil toxicity by sodium chloride-induced exopolysaccharides in *Acinetobacter* sp. strain DR1. J Biosci Bioeng 109, 118–123 (2010). 20129094 10.1016/j.jbiosc.2009.08.001

[b22] Tardy V. *et al.* Stability of soil microbial structure and activity depends on microbial diversity. Environ Microbiol Rep 6, 173–183 (2014). 24596291 10.1111/1758-2229.12126

[b23] Nacke H. *et al.* Pyrosequencing-based assessment of bacterial community structure along different management types in German forest and grassland soils. PLoS One 6, e17000 (2011). 21359220 10.1371/journal.pone.0017000 PMC3040199

[b24] Nie Y. *et al.* Diverse alkane hydroxylase genes in microorganisms and environments. Sci Rep 4, 4968 (2014). 24829093 10.1038/srep04968 PMC4021335

[b25] Hamamura N., Yeager C. M. & Arp D. J. Two distinct monooxygenases for alkane oxidation in *Nocardioides* sp. strain CF8. Appl Environ Microbiol 67, 4992–4998 (2001). 11679317 10.1128/AEM.67.11.4992-4998.2001 PMC93262

[b26] Zampolli J., Collina E., Lasagni M. & Di Gennaro P. Biodegradation of variable-chain-length *n*-alkanes in *Rhodococcus opacus* R7 and the involvement of an alkane hydroxylase system in the metabolism. AMB Express 4, 73 (2014). 25401074 10.1186/s13568-014-0073-4 PMC4230829

[b27] Radwan S. S., Sorkhoh N. A., Felzmann H. & El-Desouky A. F. Uptake and utilization of *n*-octacosane and *n*-nonacosane by *Arthrobacter nicotianae* KCC B35. J Appl Bacteriol 80, 370–374 (1996). 8849639 10.1111/j.1365-2672.1996.tb03231.x

[b28] Akbari A. & Ghoshal S. Effects of diurnal temperature variation on microbial community and petroleum hydrocarbon biodegradation in contaminated soils from a sub-Arctic site. Environ Microbiol 10.1111/1462-2920.12846 (2015). 25808640

[b29] Fierer N. *et al.* Comparative metagenomic, phylogenetic and physiological analyses of soil microbial communities across nitrogen gradients. ISME J 6, 1007–1017 (2012). 22134642 10.1038/ismej.2011.159 PMC3329107

[b30] Liu H., Xu J., Liang R. & Liu J. Characterization of the medium- and long-chain *n*-alkanes degrading *Pseudomonas aeruginosa* strain SJTD-1 and its alkane hydroxylase genes. PLoS One 9, e105506 (2014). 25165808 10.1371/journal.pone.0105506 PMC4148322

[b31] Pérez-de-Mora A., Engel M. & Schloter M. Abundance and diversity of *n*-alkane-degrading bacteria in a forest soil co-contaminated with hydrocarbons and metals: a molecular study on *alkB* homologous genes. Microb Ecol 62, 959–972 (2011). 21567188 10.1007/s00248-011-9858-z

[b32] Yergeau E., Sanschagrin S., Beaumier D. & Greer C. W. Metagenomic analysis of the bioremediation of diesel-contaminated Canadian high arctic soils. PLoS One 7, e30058 (2012). 22253877 10.1371/journal.pone.0030058 PMC3256217

[b33] Liang Y. *et al.* Functional gene diversity of soil microbial communities from five oil-contaminated fields in China. ISME J 5, 403–413 (2011). 20861922 10.1038/ismej.2010.142 PMC3105718

[b34] Jung J. *et al.* Molecular mechanisms of enhanced bacterial growth on hexadecane with red clay. Microb Ecol 70, 912–921 (2015). 25956940 10.1007/s00248-015-0624-5

[b35] Nikolopoulou M., Pasadakis N., Norf H. & Kalogerakis N. Enhanced ex situ bioremediation of crude oil contaminated beach sand by supplementation with nutrients and rhamnolipids. Mar Pollut Bull. 77, 37–44 (2013). 24229785 10.1016/j.marpolbul.2013.10.038

[b36] Bell T. H., Yergeau E., Juck D. F., Whyte L. G. & Greer C. W. Alteration of microbial community structure affects diesel biodegradation in an Arctic soil. FEMS Microbiol Ecol 85, 51–61 (2013b). 23488635 10.1111/1574-6941.12102

[b37] Hou L. *et al.* Effects of sulfamethazine on denitrification and the associated N_2_O release in estuarine and coastal sediments. Environ Sci Technol 49, 326–333 (2015). 25525860 10.1021/es504433r

[b38] Margesin R., Zimmerbauer A. & Schinner F. Monitoring of bioremediation by soil biological activities. Chemosphere 40, 339–346 (2000). 10665397 10.1016/s0045-6535(99)00218-0

[b39] Trasar-Cepedaa C., Leirósb M. C., Seoaneb S. & Gil-Sotresb F. Limitations of soil enzymes as indicators of soil pollution. Soil Biol Biochem 32, 1867–1875 (2000).

[b40] Seo H. *et al.* Complexity of cell-cell interactions between *Pseudomonas* sp. AS1 and *Acinetobacter oleivorans* DR1: metabolic commensalism, biofilm formation and quorum quenching. Res Microbiol 163, 173–181 (2012). 22202171 10.1016/j.resmic.2011.12.003

[b41] Wakeline S. A. *et al.* Structural and functional response of soil microbiota to addition of plant substrate are moderated by soil Cu levels. Biol Fertil Soils 46, 333–342 (2010).

[b42] Macdonald C. A. *et al.* Long-term impacts of zinc and copper enriched sewage sludge additions on bacterial, archaeal and fungal communities in arable and grassland soils. Soil Biol Biochem 43, 932–941 (2011).

[b43] Kandeler E., Kampichler C. & Horak O. Influence of heavy metals on the functional diversity of soil microbial communities. Biol Fertil Soils 23, 299–306 (1996).

[b44] Watanabe K., Kodama Y. & Harayama S. Design and evaluation of PCR primers to amplify bacterial 16S ribosomal DNA fragments used for community fingerprinting. J Microbiol Methods 44, 253–262 (2001). 11240048 10.1016/s0167-7012(01)00220-2

[b45] Jung J. *et al.* Change in gene abundance in the nitrogen biogeochemical cycle with temperature and nitrogen addition in Antarctic soils. Res Microbiol 162, 1018–1026 (2011). 21839168 10.1016/j.resmic.2011.07.007

[b46] Meyer F. *et al.* The metagenomics RAST server - a public resource for the automatic phylogenetic and functional analysis of metagenomes. BMC Bioinformatics 9, 386 (2008). 18803844 10.1186/1471-2105-9-386 PMC2563014

[b47] Parks D. H., Tyson G. W., Hugenholtz P. & Beiko R. G. STAMP: Statistical analysis of taxonomic and functional profiles. Bioinformatics 30, 3123–3124 (2014). 25061070 10.1093/bioinformatics/btu494 PMC4609014

[b48] Stanier R. Y., Palleroni N. J. & Doudoroff M. The aerobic pseudomonads: a taxonomic study. J Gen Microbiol 43, 159–271 (1966). 5963505 10.1099/00221287-43-2-159

[b49] Han J., Jung J., Hyun S., Park H. & Park W. Effects of nutritional input and diesel contamination on soil enzyme activities and microbial communities in Antarctic soils. J Microbiol 50, 916–924 (2012). 23274977 10.1007/s12275-012-2636-x

[b50] Singh B. K. *et al.* Loss of microbial diversity in soils is coincident with reductions in some specialized functions. Environ Microbiol 16, 2408–2420 (2014). 24422656 10.1111/1462-2920.12353

[b51] Miki T., Yokokawa T. & Matsui K. Biodiversity and multifunctionality in a microbial community: a novel theoretical approach to quantify functional redundancy. Proc Biol Sci 281, 20132498 (2014). 24352945 10.1098/rspb.2013.2498 PMC3871314

