# Supplementary material for: Metagenomic and functional analyses of the consequences of reduction of bacterial diversity on soil functions and bioremediation in diesel-contaminated microcosms
Source: Sci Rep. 2016 Mar 14;6:23012. doi: 10.1038/srep23012 (PMC4789748; doi:10.1038/srep23012)

**Supplementary Information**

**Metagenomic and functional analyses of the consequences of reduction of bacterial diversity on soil functions and bioremediation in diesel-contaminated microcosms**

Jaejoon Jung[a], Laurent Philippot[b], Woojun Park[a]

[a]Laboratory of Molecular Environmental Microbiology, Department of Environmental Science and Ecological Engineering, Korea University, Seoul, 02841, Republic of Korea

[b]INRA Dijon, UMR 1347 Agroecologie, Dijon, France

**Contributions:** JJ and WP designed the study. JJ performed all experiments and analyses. JJ wrote the first draft of the manuscript. LP and WP provided substantial discussion and modifications. All authors contributed to the final version of the manuscript and approved it.

**Competing financial interests:** The authors declare no competing financial interests.

**Running title:** Loss of diversity results in trade-offs between ecological processes

***Corresponding author:** Dr. Woojun Park, Department of Environmental Science and Ecological Engineering, Korea University, Seoul, 02841, Republic of Korea

**E-mail:** wpark@korea.ac.kr

**Fax:** +80-2-953-0737

**Phone:** +82-2-3290-3067

**Supplementary Table S1** Result of metagenomic sequence

| Sample | Dilution | Base pairs | Reads | Contig | Average length (bp) |
|---|---|---|---|---|---|
| Control | $10^{-2}$ | 391,704,381 | 8,854,928 | 175,757 | 2,229 |
| | $10^{-5}$ | 82,434,475 | 5,357,854 | 16,901 | 4,877 |
| Diesel | $10^{-2}$ | 190,340,275 | 6,397,443 | 74,532 | 2,554 |
| | $10^{-5}$ | 64,392,817 | 6,316,091 | 19,043 | 3,381 |
| Diesel+Red clay | $10^{-2}$ | 241,772,807 | 8,455,484 | 110,118 | 2,196 |
| | $10^{-5}$ | 81,513,354 | 7,631,667 | 25,411 | 3,208 |
| Diesel+Processed | $10^{-2}$ | 167,432,219 | 8,371,381 | 44,196 | 3,788 |
| red clay | $10^{-5}$ | 76,697,091 | 7,956,337 | 19,412 | 3,951 |

**Supplementary Fig. S1.** Alpha diversity of differentially inoculated microcosms with different condition.

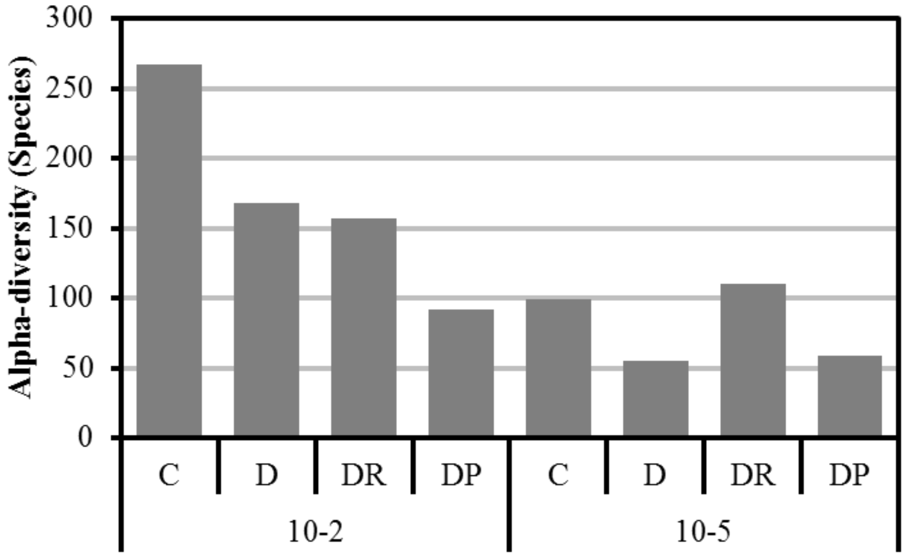

**Supplementary Fig. S2.** Taxonomic affiliation of functional genes from metagenomic sequence data analyzed by MG-RAST. (a) Domain (b) Phylum (c) Genus

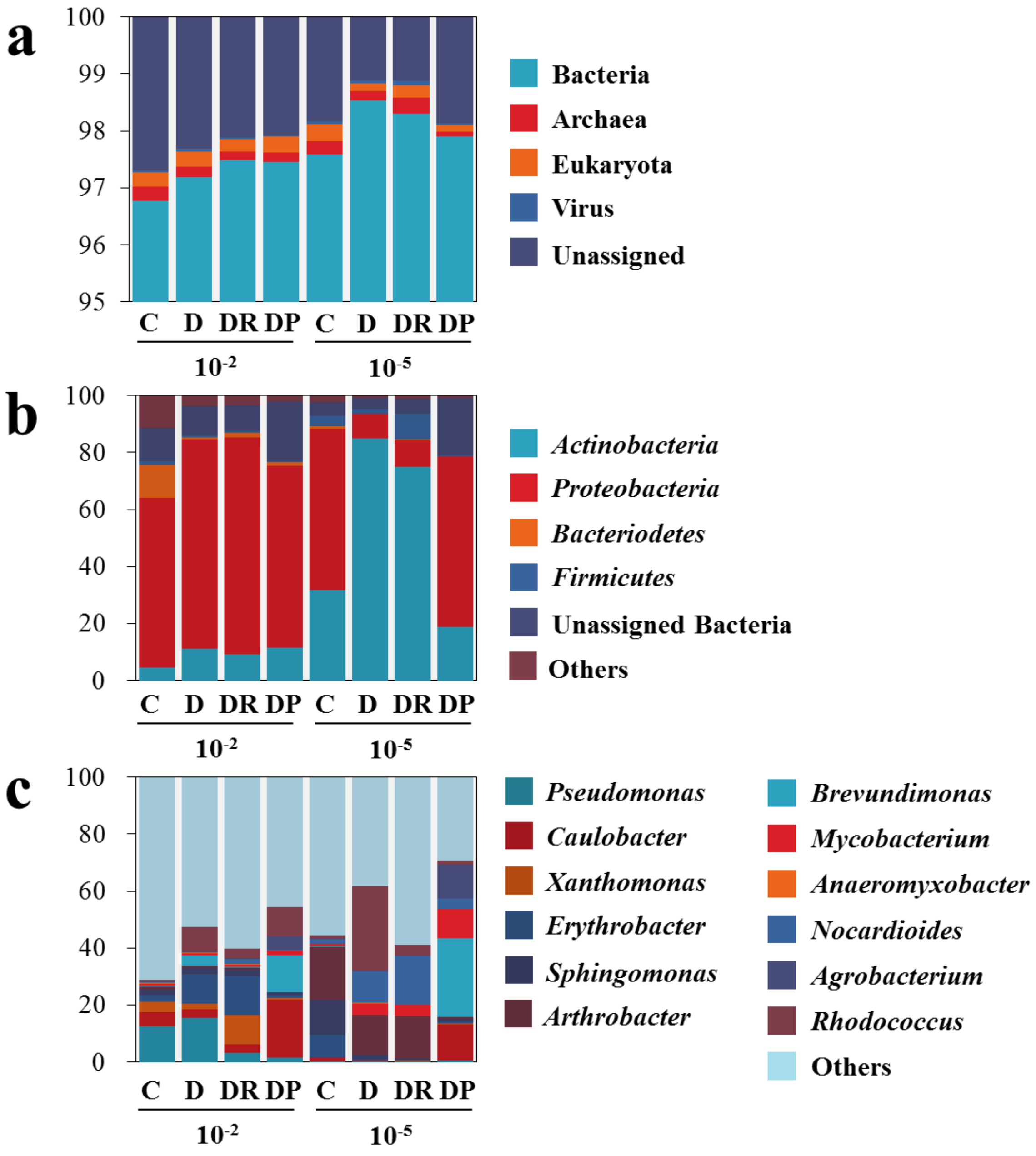

**Supplementary Fig. S3.** Principle component analysis of (A) Community structure at phylum level and (B) functional genes determined by metagenomic data (KEGG Orthology level 3 category).

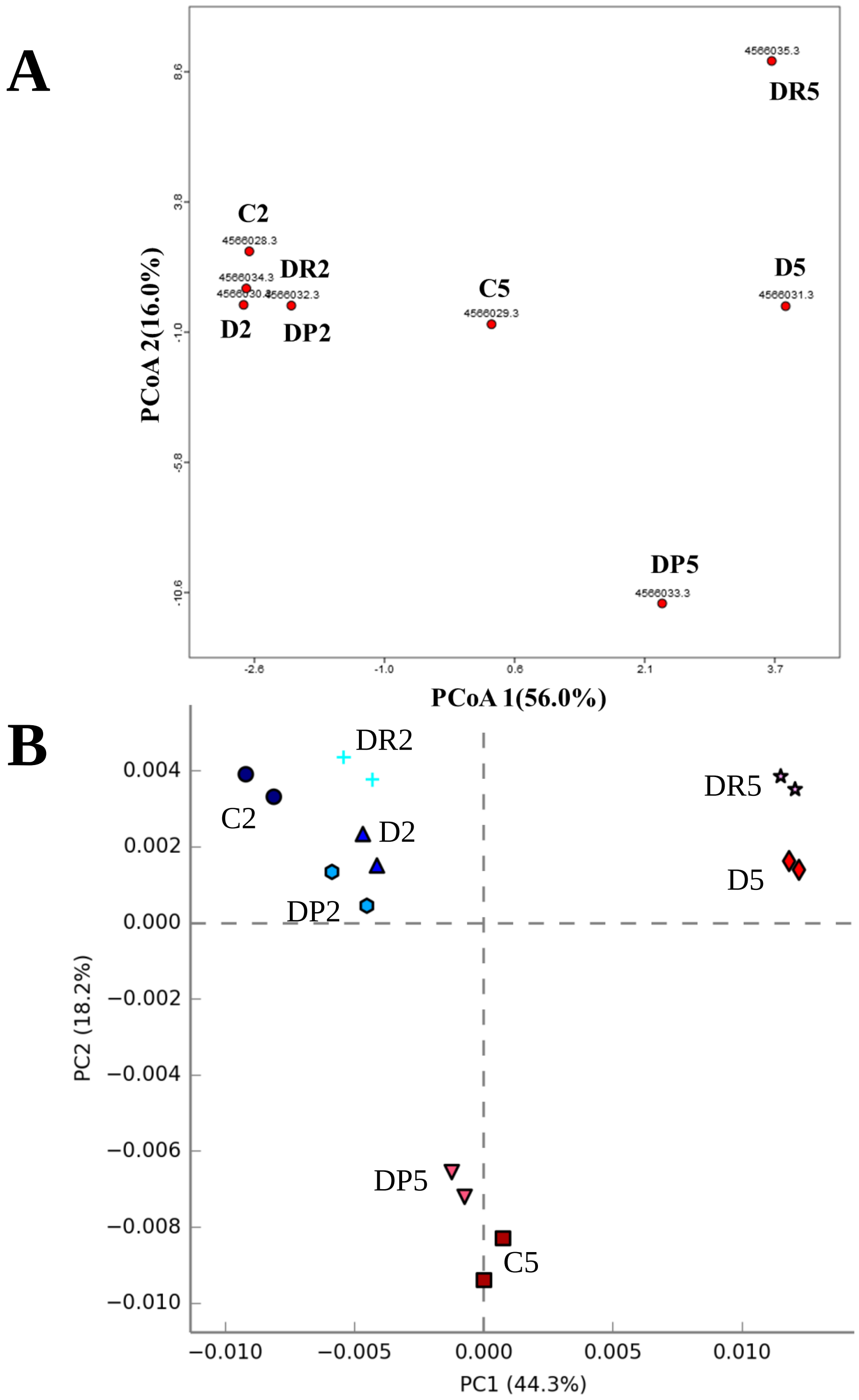

**Supplementary Fig. S4.** Measurement of various soil enzyme activities related to nutrient cycling (a) and the phylogenetic analysis of the related genes (b)

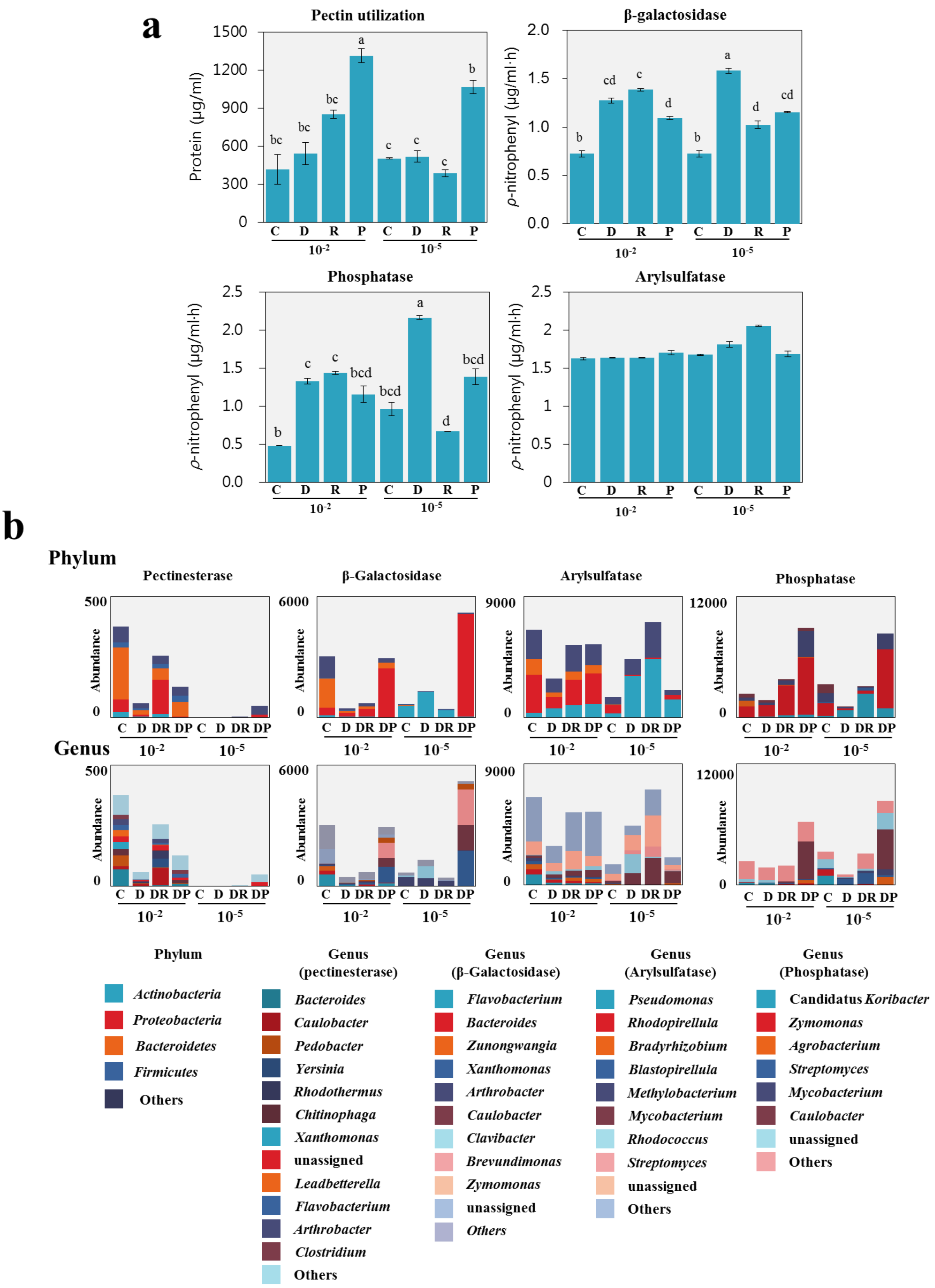

Supplement: Supplementary Information [file srep23012-s1.pdf]
